# Supplementary material for: The role of gene duplication and paralog specialisation in the evolution of the mammalian PRPS complex
Source: Nat Commun. 2025 Jul 8;16:6076. doi: 10.1038/s41467-025-61216-z (PMC12238573; doi:10.1038/s41467-025-61216-z)
Supplement: Supplementary file 1 — Supplementary Information File [file 41467_2025_61216_MOESM1_ESM.pdf]

SUPPLEMENTARY INFORMATION for

The role of gene duplication and paralog specialisation in the evolution of the mammalian PRPS complex

AUTHORS: Bibek R. Karki[1], Austin C. MacMillan[1], Sara Vicente-Muñoz[2], Kenneth D. Greis[1], Lindsey E. Romick[2], John T. Cunningham[1*]

Corresponding author: cunnijn@ucmail.uc.edu

The file contains:

Supplementary Figures 1–10 and Supplementary Table 1

Supplementary Figure 1

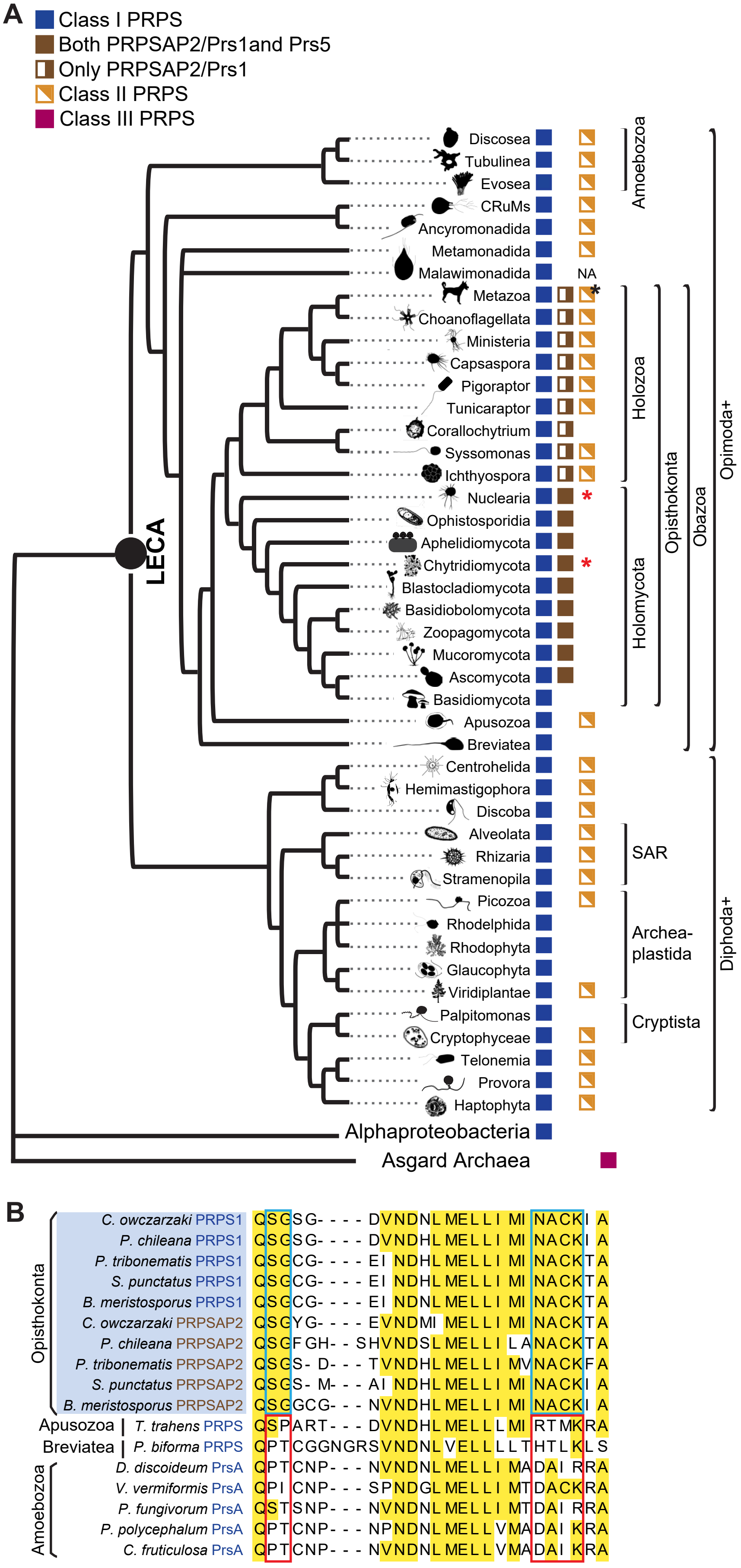

Supplementary Figure 1. PRPS homolog distribution in an alternative phylogenetic tree and emergence of Opisthokonta PRPSAP2 from Opisthokonta PRPS1

A Phylogenetic distribution of PRPS homologs in eukaryotes and PRPS homologs containing NHR in opisthokonts (presence/absence) are noted across this recent alternative phylogenetic tree adapted from Williamson et. al[30]. This tree, derived using mitochondrial proteins of alphaproteobacterial origin, indicates that the eukaryotic root is positioned between two major multi-supergroup clusters, termed ‘Opimoda+’ and ‘Diphoda+’. PRPSAP2 represent orthologs of mammalian PRPSAP2 while Prs1 and Prs5 represent orthologs of *S. cerevisiae* Prs1 and Prs5, respectively. A black asterisk next to Metazoan Class II PRPS enzymes indicates their presence in most metazoans, except Craniata. Red asterisks denote that Class II PRPS enzymes have been identified in only two species from Nuclearia and one species from Chytridomycota. Alphaproteobacteria, the primary source of mitochondrial genes during eukaryogenesis, possess Class I PRPS but not Class II PRPS (Supplementary Data 1). Asgard archaea, the closest archaeal relative of eukaryotes possess overwhelmingly Class III-like PRPS but not Class II PRPS (see Figshare datasets). **B** N-terminal residues from a sequence alignment of PRPS homologs from representative organisms in Amorphea. Based on sequence identity, Opisthokonta PRPSAP2 sequences are more similar to Opisthokonta PRPS enzymes (blue box) than to PRPS from Apusozoa, Breviatea and Amoebozoa (red box) indicating that PRPSAP2 likely emerged from ancestral Opisthokonta PRPS1.

Supplementary Fig. 2

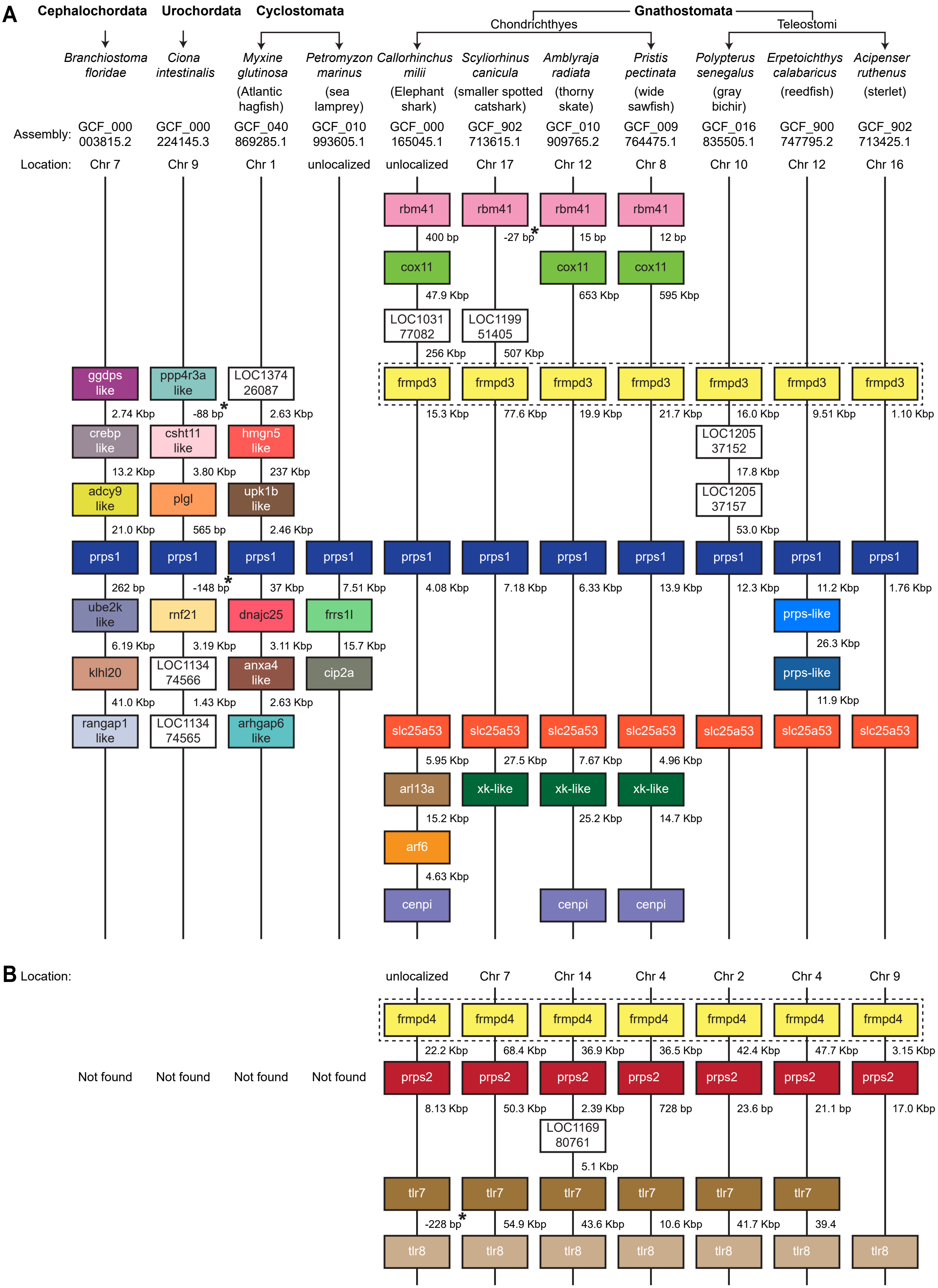

Supplementary Figure 2. Gene synteny comparisons indicate that PRPS2 duplication from PRPS1 in jawed Vertebrata likely occurred during the second round of whole genome duplication in Vertebrates (2R).

A, B Genomic regions surrounding *prps1* and *prps2*, respectively in representative species from Cephalochordata, Urochordata, Cyclostomata and basal Gnathostomata. The chromosome containing the syntenic region and the corresponding RefSeq genome assembly are shown at the top for each species. Each gene is represented by a colored box, while uncharacterized genes are labeled with their GeneID and shown in white. The figure was not drawn to scale, but the number of intergenic base pairs (bp) was indicated. Black asterisks indicate regions with overlapping genes, where the extent of overlap is shown as a negative value. The *prps1* and *prps2* paralogs and their neighboring genes demonstrate syntenic genomic locations in jawed Vertebrates, but synteny is lacking among Cephalochordata, Urochordata, and Cyclostomata. Notably, in jawed Vertebrates, *frmpd3*, located adjacent to *prps1*, has a duplicated paralog, *frmpd4*, situated next to *prps2* (highlighted by dashed boxes). This suggests that *prps2* originated from the duplication of an ancestral chromosomal fragment harboring *prps1* likely during the second round of whole genome duplication in jawed vertebrates (2R).

Supplementary Fig. 3

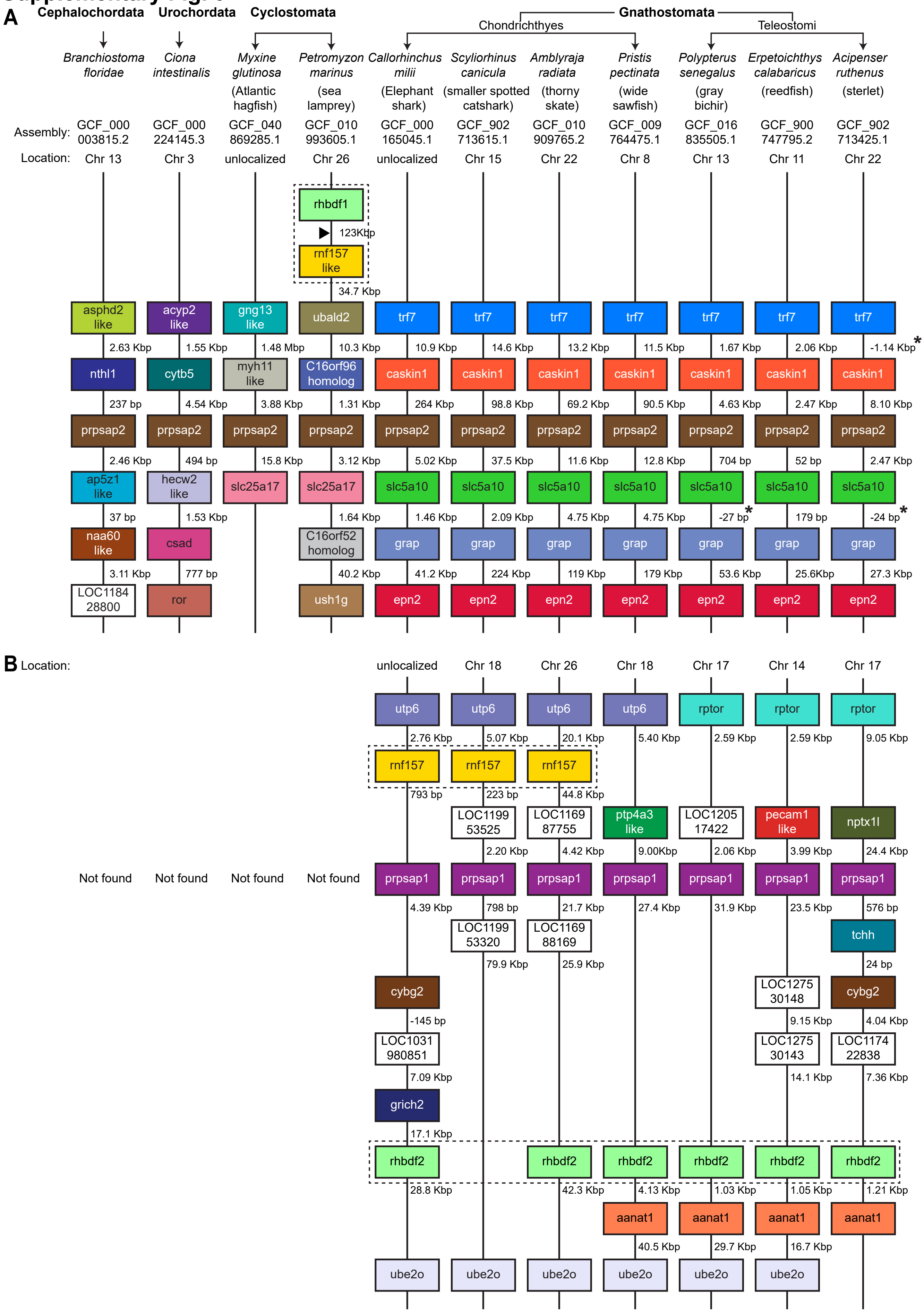

Supplementary Figure 3. Gene synteny comparisons indicate that PRPSAP1 duplication from PRPSAP2 in jawed Vertebrata likely occurred during the second round of whole genome duplication in Vertebrates (2R).

A, B Genomic regions surrounding *prpsap2* and *prpsap1*, respectively in representative species from Cephalochordata, Urochordata, Cyclostomata and basal Gnathostomata. The chromosome containing the syntenic region and the corresponding RefSeq genome assembly are shown at the top for each species. Each gene is represented by a colored box, while uncharacterized genes are labeled with their GeneID and shown in white. The figure was not drawn to scale, but the number of intergenic base pairs (bp) was indicated. Black asterisks indicate regions with overlapping genes, where the extent of overlap is shown as a negative value. A black triangle between *rhbdf1* and *rnf157-like* marks a gap where six additional genes are present but not shown. The *prpsap2* and *prpsap1* paralogs and their neighboring genes demonstrate syntenic genomic locations in jawed Vertebrates, but synteny is lacking among Cephalochordata, Urochordata, and Cyclostomata. Notably, *rhbdf1* and *rnf157-like* genes located in the vicinity of *prpsap2* in Cyclostomata (sea lamprey) have duplicated paralogs *rhbdf2* and *rnf157* around *prpsap1* in Gnathostomata (elephant shark, smaller spotted shark, and thorny skate) (highlighted by dashed boxes). This suggests that *prpsap1* originated from the duplication of an ancestral chromosomal fragment harboring *prpsap2* likely during the second round of whole genome duplication in jawed vertebrates (2R).

Supplementary Fig. 4

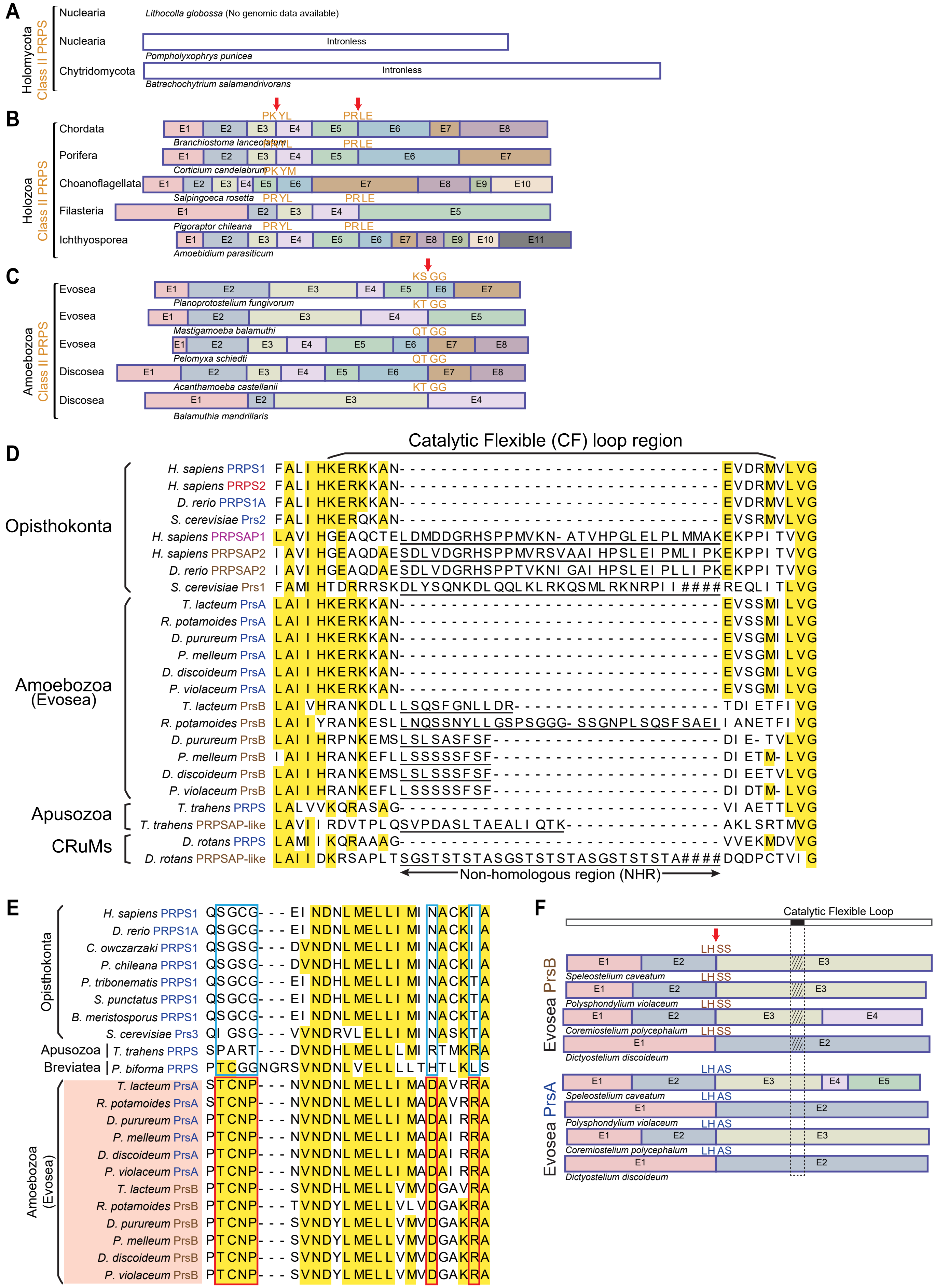

Supplementary Figure 4. Independent Gene Duplications of PRPS encoding genes resulted in PRPS paralogs with expanded CF loops across Amorphea.

A Among the three identified Holomycota species harboring Class II PRPS, *P. punicea* and *B. salamandrivorans* lack introns, suggesting potential horizontal transfer. *L. globossa* (Nuclearia) may have acquired Class II PRPS similarly, though incomplete/non-comprehensive genomic data prevents confirmation of intron status. **B, C** Analysis of splice site junctions among Class II PRPS across different representative organisms in Holozoa and Amoebozoa. Gene structures shown with exons as uniquely colored boxes; introns not displayed. Multiple sequence alignment (MSA) of translated PRPS sequences via Clustal Omega highlights conserved splice site junctions (red arrows), with the surrounding amino acids shown. This supports lineage-specific ancestry of Class II PRPS in Holozoa and Amoebozoa. **D** Sequence alignments of CF loop and flanking regions from PRPS homologs across representative organisms in Opisthokonta, Amoebozoa (Evosea), Apusozoa, and CRuMs. Evosea PrsA represents the ancestral enzyme; PrsB features CF loop insertions (NHRs) similar to Opisthokonta PRPSAP2. PRPS homologs from Apusozoa and CRuMs sharing these insertions are termed "PRPSAP-like". NHRs are underlined. For *S. cerevisiae* Prs1 and *D. rotans* PRPSAP-like, additional insertions shown with hatch marks. **E** N-terminal alignment of PRPS homologs from Amorphea. Based on sequence identity, Evosea PrsB sequences share higher identity with Evosea PrsA (red box) than with PRPS from Apusozoa, Breviatea, or Opisthokonta (blue box) supporting PrsB's origin via duplication of ancestral Evosea PrsA. **F** Analysis of splice site junctions of PrsA and PrsB across Evosea. Gene structures shown with exons as uniquely colored boxes; introns not displayed. MSA highlights conserved splice junction (red arrow) with flanking amino acids. Top bar shows full *S. caveatum* PRPS1, with CF loop indicated by black boxes. Dotted lines project CF loop positions onto homologs below. PrsB insertions in CF loops denoted by hatch marks (not to scale; NHR lengths variable).

Supplementary Fig. 5

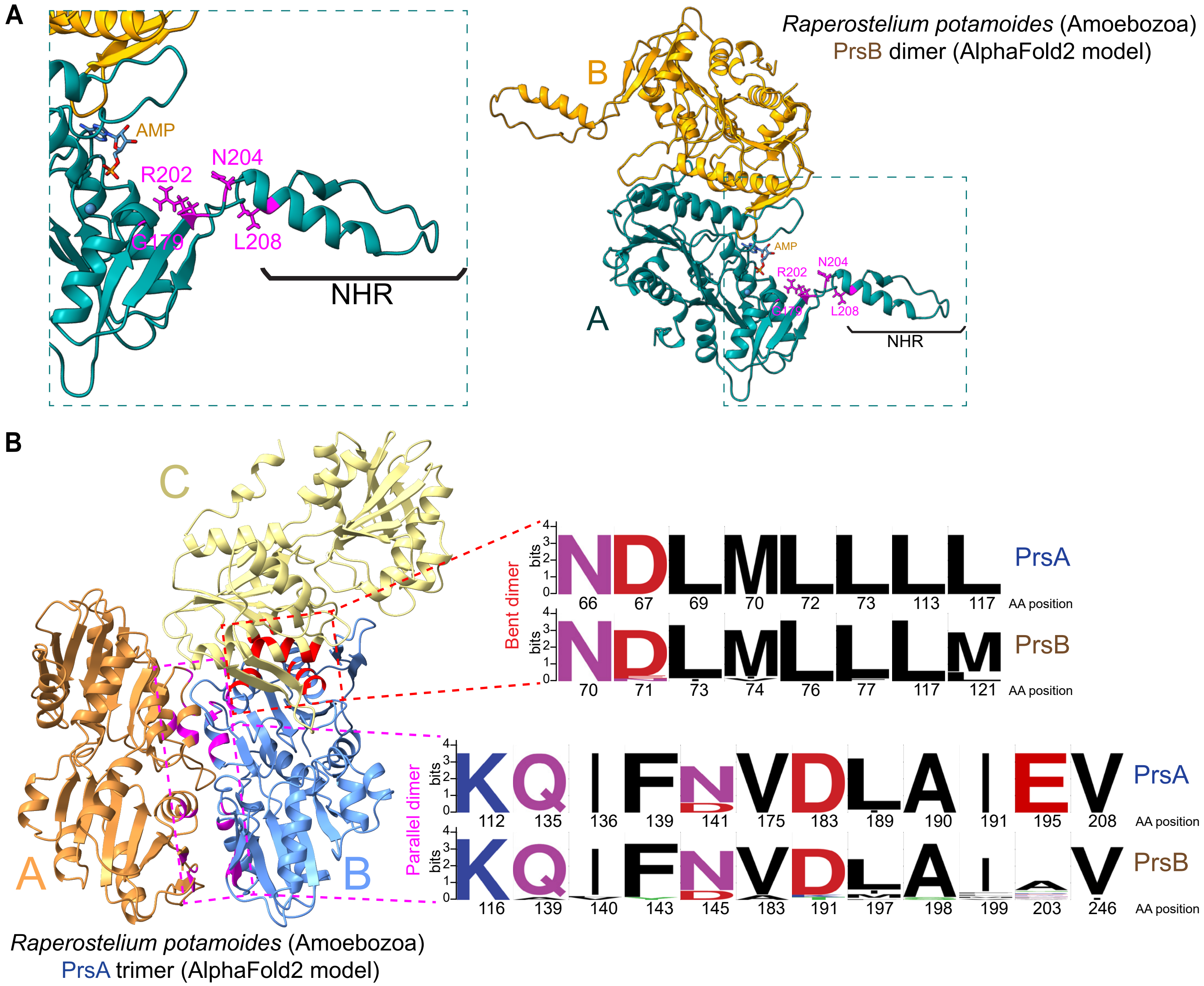

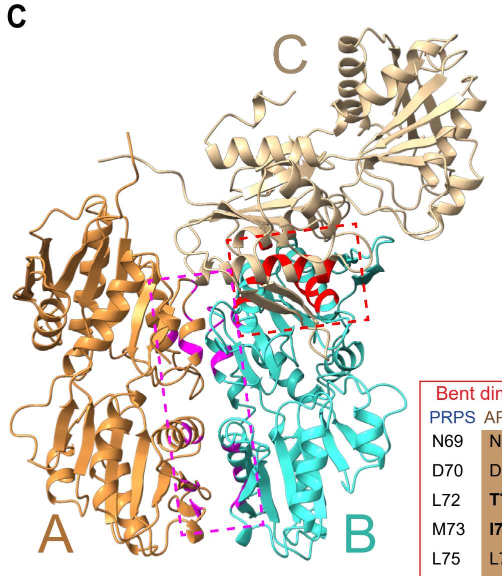

Thecamonas trahens (Apusozoa)
PRPS trimer (AlphaFold2 model)

Bent dimer	
PRPS	AP-like
N69	N70
D70	D71
L72	**T73**
M73	**I74**
L75	L76
L76	L77
D116	D118
I120	L122

Parallel dimer	
PRPS	AP-like
S115	**A117**
Q138	Q140
I139	I141
F142	F144
T144	S146
V181	V186
N189	**A194**
L200	L205
A201	A206
L202	V207
Q206	**D211**
V219	V240

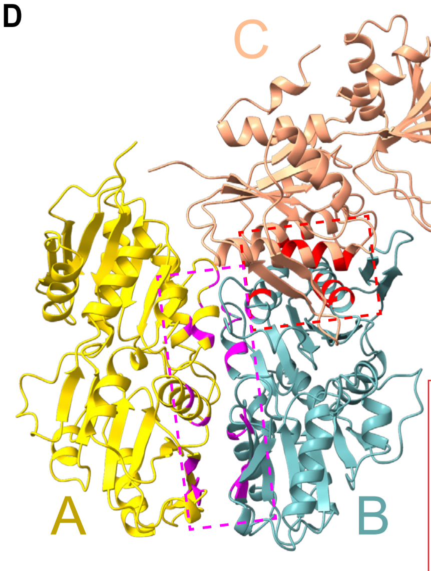

Diphylleia rotans (CRuMs)
PRPS trimer (AlphaFold2 model)

Bent dimer	
PRPS	AP-like
N65	N65
E66	D66
L68	V68
M69	M69
L71	L71
F72	L72
D112	**L112**
L116	L116

Parallel dimer	
PRPS	AP-like
S111	**K111**
Q134	Q134
I135	I135
F138	F138
Q140	**D140**
V175	V180
D183	**A188**
L195	L194
A196	A195
M197	**I196**
Q201	R200
V214	I294

Supplementary Figure 5. PRPSAP-like homologs in non-opisthokont Amorphea lineages are likely non-catalytic with conserved dimer interfaces.

A Predicted dimeric structure of *R. potamoides* PrsB (annotated from SRX8374346-9) from the AlphaFold2 model, and a zoom in highlights four non-conserved residues in PrsB (magenta) at the corresponding positions of active site residues for PrsA (Supplementary Table 1). AMP modeled into the dimer to denote the putative ATP binding site. NHR represents the insertion in the CF loop. **B** Predicted trimeric structure of *R. potamoides* PrsA (annotated from SRX8374346-9) from the AlphaFold2 model. In the dashed box, red- and magenta-colored residues represent those involved in the formation of bent (B and C subunits) and parallel (A and B subunits) dimers, respectively. Amino acid sequence of *B. subtilis* PRPS aligned with the Amoebozoa PRPS homologs, and the corresponding dimer interface residues selected for generating the WebLogo. Sequences for PrsA (n = 19) and PrsB (n = 20) derived from representative organisms within Amoebozoa. The numbers below the logo sequences indicate the corresponding residues positions of *R. potamoides* PrsA and PrsB. Significant sequence conservation between PrsA and PrsB indicates the potential for heteromeric associations. **C, D** represents the predicted trimeric structure of *T. trahens* (representative organism from Apusozoa) PRPS (XP_013753676.1) in (C) and *D. rotans* (representative organism from CRuMs) PRPS (annotated from SRX3153023) in (D) from the AlphaFold2 model. Red and magenta residues in the dashed box indicate those forming bent (B and C) and parallel (A and B) dimers, respectively. A comparison of dimer interface residues at similar positions in PRPSAP-like is shown. Non-conserved residues in PRPSAP-like relative to PRPS are shown in bold.

Supplementary Fig. 6

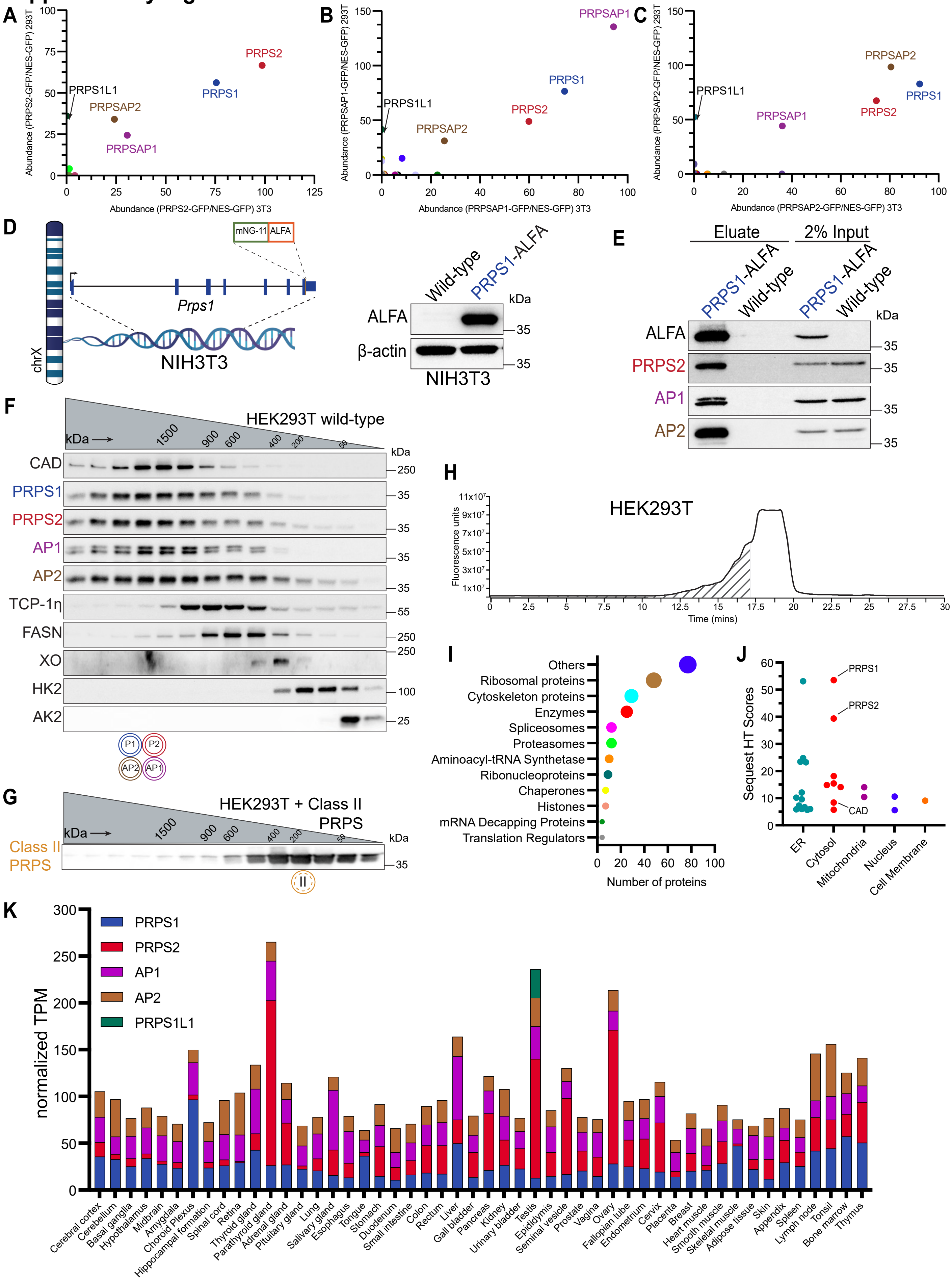

Supplementary Figure 6. The stable Class I PRPS complex is one of the largest cytosolic metabolic assemblies in cells.

A-C Scatter plots from mass spectrometry (MS) runs of eluates from GFP IP in stably expressing PRPS2-GFP, PRPSAP1-GFP, and PRPSAP2-GFP, respectively in NIH3T3 (x-axis) and HEK293T (y-axis) cells. Axes represent square root-transformed SEQUEST HT scores normalized to control. **D** Schematic of *Prps1* gene with an endogenous ALFA tag knocked in frame at the C-terminus. mNG-11 represents the eleventh beta-strand of the split monomeric Neon Green protein and ALFA is an epitope tag containing residues – PSRLEEELRRRLTEP[84]. Western blot validating full-length expression of the endogenously tagged PRPS1-ALFA protein is shown. **E** ALFA pulldown from the whole cell extracts of knock-in NIH3T3 cells demonstrating the interaction of endogenous PRPS1 with PRPS2, PRPSAP1, and PRPSAP2. **F, G** Western blot analysis of SEC fractions collected from HEK293T native whole cell lysates (F) and HEK293T cells transiently transfected with *B. lanceolatum* Class II PRPS-ALFA (G). Cell lysates were fractionated on a Superose 6 Increase 3.2/300 column. Immunoblots probing PRPS complex members and internal standards are shown. In the pictogram, double circle means multiple copies of the protein are interacting within the heteromeric complex and double circle with dotted inner circle means multiple copies of the protein are forming homo-oligomers. **H** Chromatogram showing SEC traces of HEK293T whole cell lysates fractionated on a Bio SEC-5 2000Å column, which offers better resolution for proteins/protein complexes in high molecular weight (HMW) range. Proteins that eluted in the fractions shown as hatch marks were sent for mass-spectrometry analyses for identification of proteins in HMW range. **I** Classification of HMW proteins based on their functions (manual curation) from the mass-spectrometry dataset obtained from fractions collected in (H). **J** Subclassification of enzymes from (I) based on their cellular localization. **K** Normalized transcript per million (nTPM) of PRPS complex components in various human tissues obtained from Human Protein Atlas. Western blot data (E-G) are representative of at least 2 biological repeats. Source data are provided as a Source Data file.

Supplementary Fig. 7

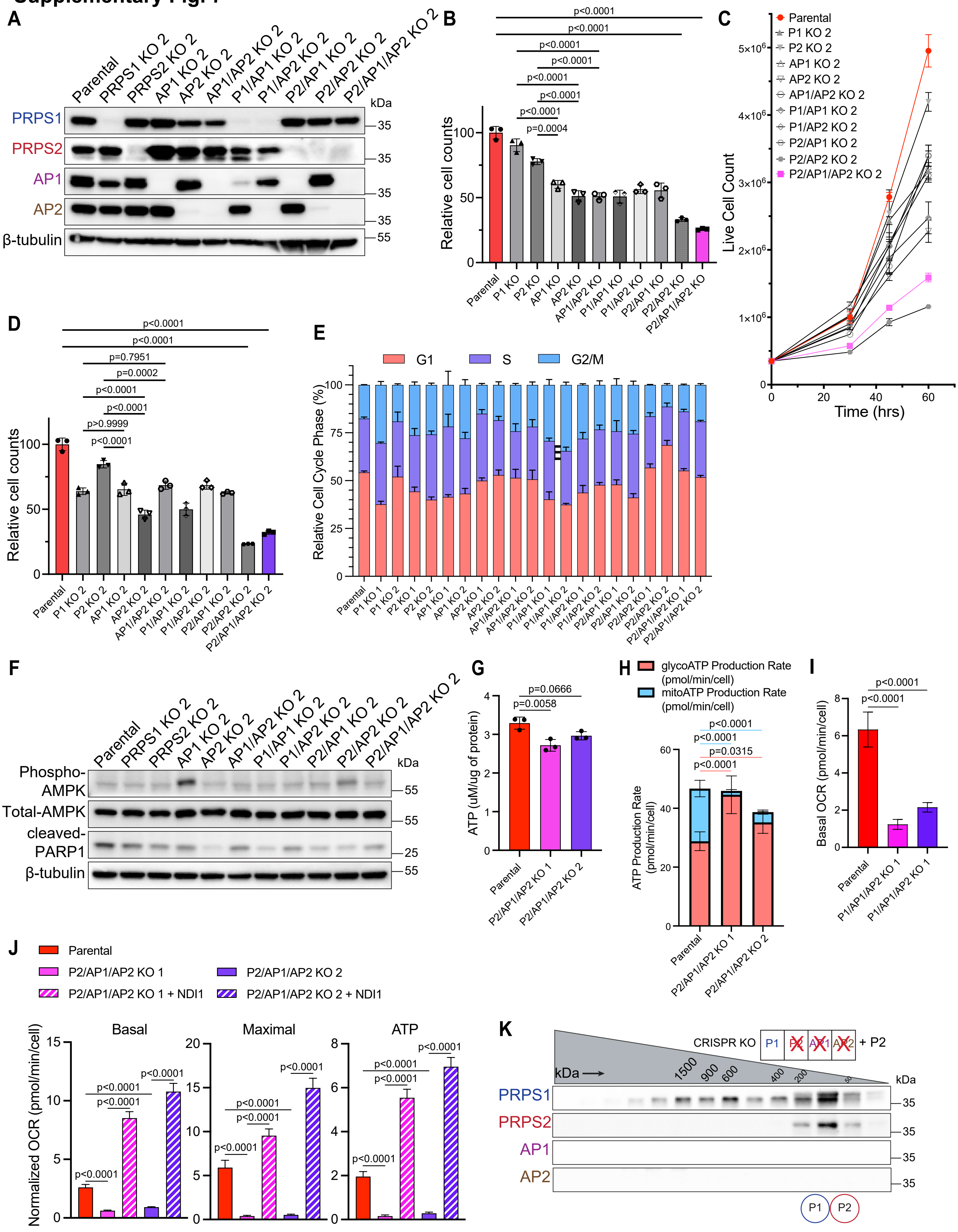

Supplementary Figure 7. Additional phenotypic characterization of NIH3T3 isogenic knockout-lines

A Western blot validation of the second set of CRISPR-Cas9-generated isogenic knockout NIH3T3 cell lines. **B** Relative cell counts at 60 hour timepoints comparing cell lines shown in Fig.4B. **C** Proliferation for cell lines generated in (A) (n = 3 technical replicates). **D** Relative cell counts at 60 hour timepoints comparing cell lines shown in (C). **E** Bar graph depicting propidium-iodide-based cell cycle profiles: G1, S, and G2/M phases for the panel of NIH3T3 knockout cell lines (n = 3 technical replicates). **F** Immunoblots probed with phospho-AMPK (T172) and cleaved PARP1 antibody in the panel of NIH3T3 parental and knockout cell lines as readouts for energy stress and apoptosis, respectively. **G** Total cellular ATP (normalized to the protein content) measured by ATP determination assay in NIH3T3 parental and P2/AP1/AP2 KO cell lines (n = 3 experimental replicates). **H** ATP production rate determined by Seahorse ATP Rate assay in NIH3T3 parental and P2/AP1/AP2 KO cell lines (n = 8 technical replicates). **I** Quantification of basal respiration measured by Seahorse ATP Rate assay in NIH3T3 parental and P2/AP1/AP2 KO cell lines (n = 8 technical replicates). **J** Quantification of basal respiration, maximal respiration (post FCCP injection), and respiration coupled to ATP production measured by Seahorse ATP Rate Assay in NIH3T3 parental and NDI1 expressing P2/AP1/AP2 KO cell lines (n = 7 technical replicates). **K** Western blot analysis of SEC fractions collected from NIH3T3 P2/AP1/AP2 KO native whole cell lysates transiently transfected with PRPS2. Cell lysates were fractionated on a Superose 6 Increase 3.2/300 column. In the pictogram below SEC immunoblots, a single circle means a single protein is interacting within the complex. Western blot data (D, H) are representative of at least 2 biological repeats. Data are represented as mean ± SD for (B-E, G-J). Statistical comparisons made using one-way ANOVA followed by Tukey's HSD post hoc test (B, D, G-J). P values < 0.05 are not significant. Source data are provided as a Source Data file.

Supplementary Fig. 8

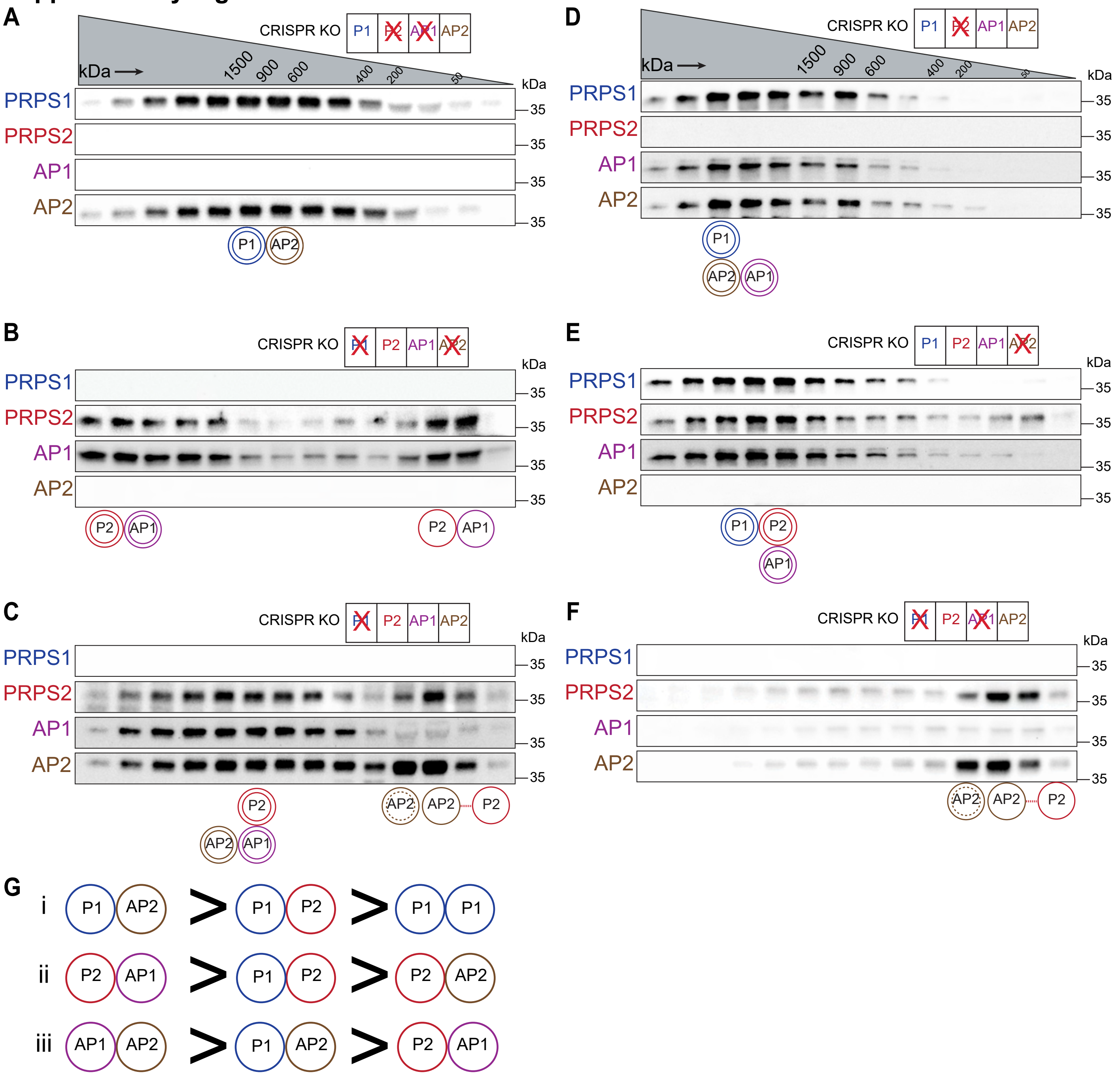

Supplementary Figure 8. SEC profiles of additional isogenic knockout lines and dimeric pairing preferences

A-F Western blot analysis of SEC fractions collected from native whole cell lysates of NIH3T3 P2/AP1 KO cells (A), P1/AP2 KO cells (B), P1 KO cells (C), P2 KO cells (D), AP2 KO cells (E), and P1/AP1 KO cells (F). Cell lysates were fractionated on a Superose 6 Increase 3.2/300 column. Circular pictograms below SEC immunoblots schematize PRPS complex configurations. Double circle denotes multiple copies of the protein interacting in a heteromeric complex. Double circle with dotted inner circle denotes multiple copies forming homo-oligomers. Single circle denotes a single protein interacting within the complex. Dotted line joining two proteins indicates weak interaction. **G** Dimeric pairing preferences among PRPS complex members determined by experimental data (i, ii) and inferred predictions (iii). Western blot data (A-F) are representative of at least 2 biological repeats. Source data are provided as a Source Data file.

Supplementary Fig. 9

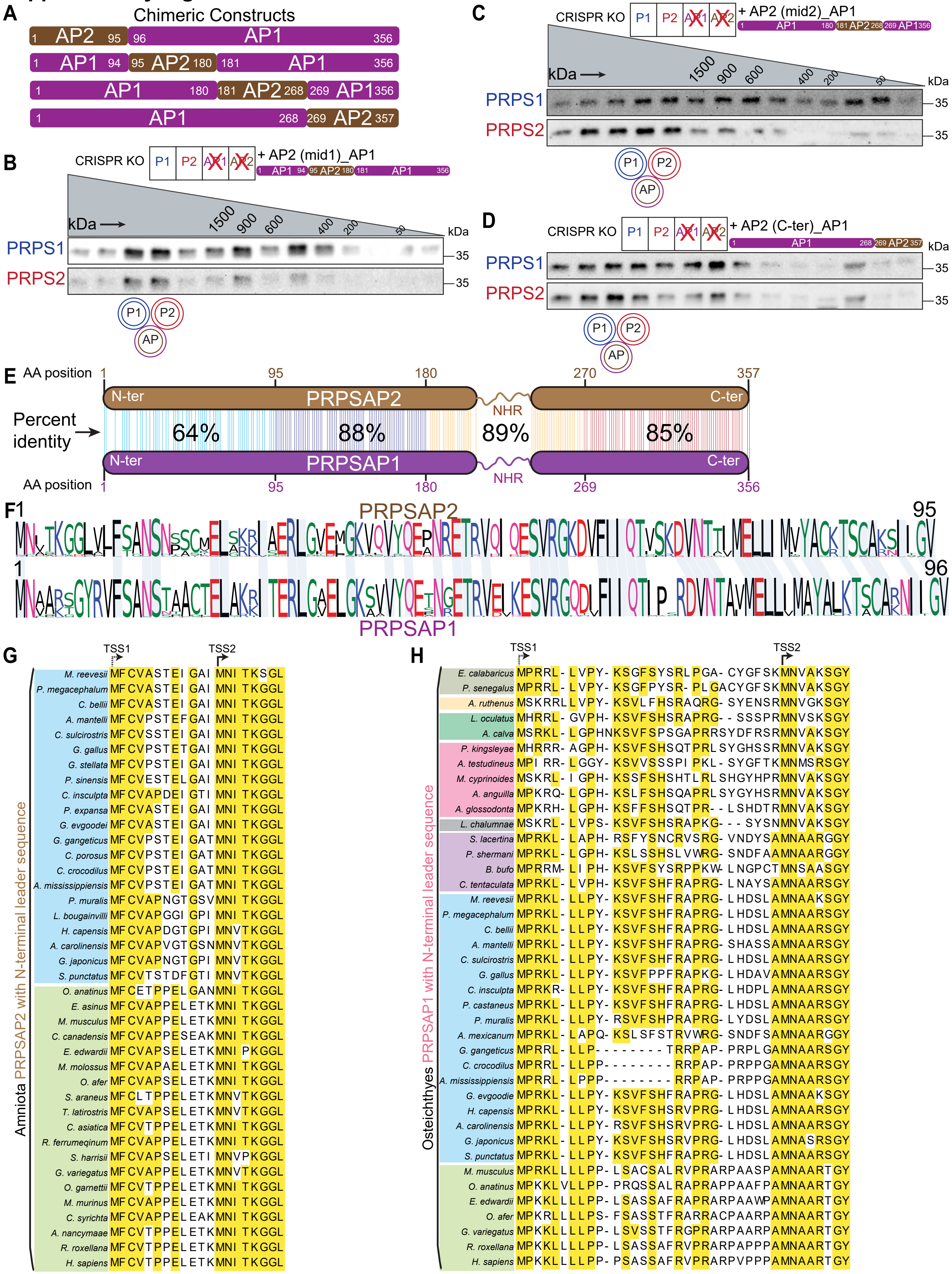

Supplementary Figure 9. N-terminus of PRPSAPs govern complex assembly

A Schema of chimeric constructs created to test AP1-specific domains that confer complex elongation property. **B-D** Western blot analysis of SEC fractions collected from native whole-cell lysates of NIH3T3 AP1/AP2 KO cells stably expressing chimeric AP1 constructs: one containing residues 95-180 from AP2 (B), residues 181-268 from AP2 (C), and AP2's C-terminus (residues 269-357) (D). **E** Comparison of human AP1 and AP2 amino acid sequence. Four regions switched (as indicated in Supplementary Fig.9A) are highlighted with different colors. Each line connecting AP1 and AP2 represents identical amino acids at that position. Regions spanning residues 1-95 show the greatest variation, with only 64% amino acid identity. Poorly conserved, highly variable amino acids present in the NHRs were excluded from this analysis. **F** MetaLogo depicting the multiple sequence alignment of the N-terminal amino acid residues of AP1 and AP2 from representative organisms in jawed Vertebrata (n = 92 for AP1 and n = 93 for AP2). Residue numbers for AP1 and AP2 correspond to the human homologs (AAH09012.1 and NP_001340030.1, respectively). **G, H** N-terminal residues from a sequence alignment of PRPSAP2 (G) and PRPSAP1 (H) from representative organisms in Amniota and Osteichthyes, respectively. TSS1 and TSS2 represents the upstream and downstream translation start sites, respectively. Cell lysates were fractionated on a Superose 6 Increase 3.2/300 column. Circular pictograms below SEC immunoblots schematize PRPS complex configurations. Double circle denotes multiple copies of the protein interacting in a heteromeric complex. Western blot data (B-D) are representative of at least 2 biological repeats. Source data are provided as a Source Data file.

Supplementary Fig. 10

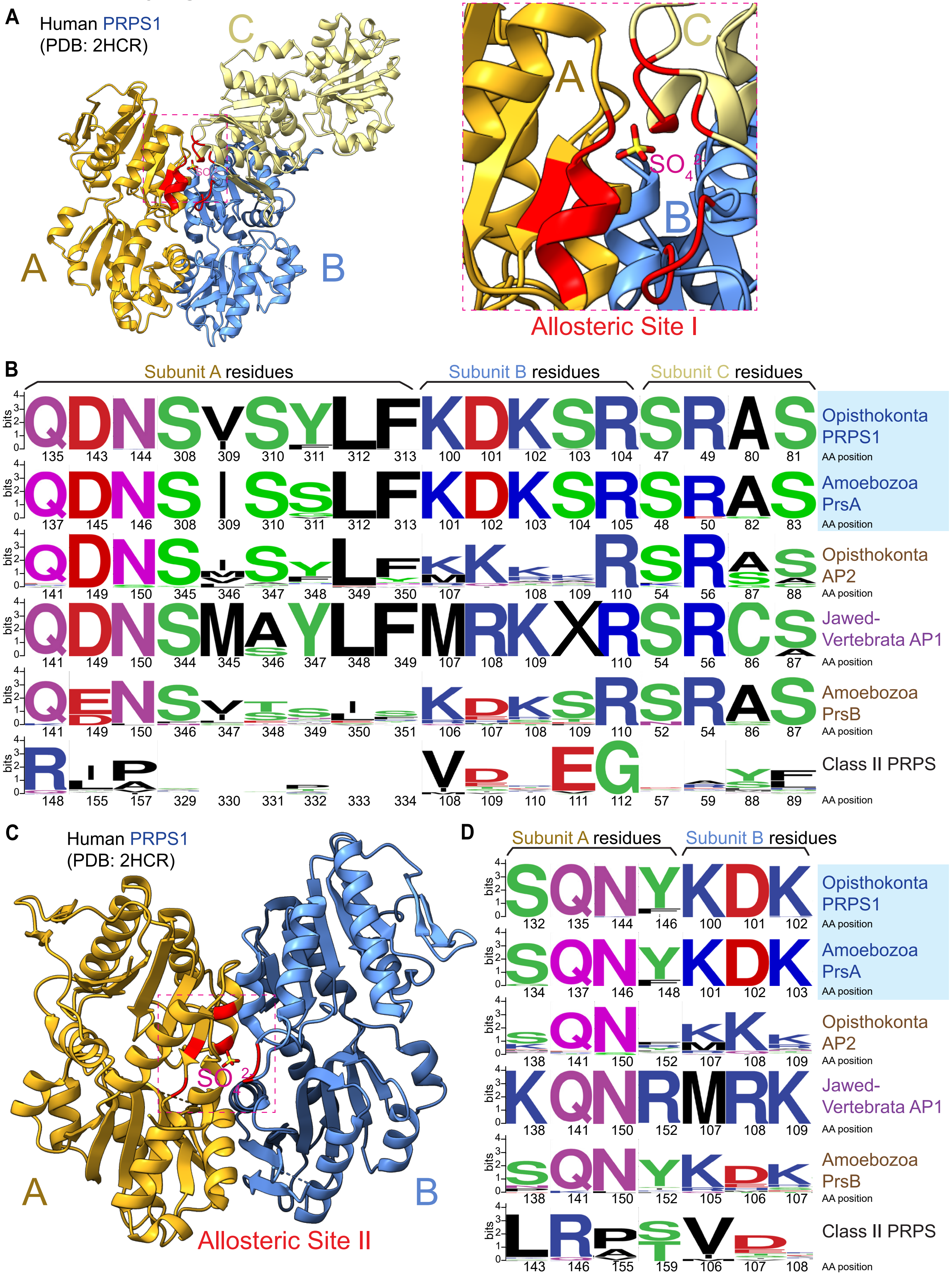

Supplementary Figure 10. Comparison of allosteric site across PRPS homologs

A Structure of trimeric human PRPS1 (PDB: 2HCR). Dashed box represents the allosteric site I, a zoom in of this site shows SO_4^2 (represents phosphate of ADP) positioned at the trimeric interface and red color indicates residues from each subunit contributing to the formation of allosteric site I. **B** Amino acid sequence of human PRPS1 was aligned with sequences of representative organisms from Opisthokonta PRPS1 (n = 44), Amoebozoa PrsA (n = 19), Opisthokonta PRPSAP2 (n = 46), jawed Vertebrata PRPSAP1 (n = 92), Amoebozoa PrsB (n = 20) and Class II PRPS (n = 53), and corresponding allosteric site I residues were selected for generating the WebLogo. Numbers below the logo sequences indicate the corresponding residue positions of human PRPS1 and PRPSAP2 (for Opisthokonts), *R. potamoides* PrsA and PrsB (for Amoebozoa), human PRPSAP1 (for jawed Vertebrata), and *A. parasiticum* Class II PRPS (annotated from SRX179384). X represents an absence of amino acid residue at that position in jawed Vertebrata AP1. Class II PRPS enzymes are shown as a comparator since they lack allosteric sites found in Class I PRPS. **C** Structure of dimeric human PRPS1 (PDB: 2HCR). Dashed box represents the allosteric site II, a zoom in of this site shows SO_4^2 positioned at the dimer interface and red color indicates the residues from each subunit contributing to the formation of allosteric site II. **D** Amino acid sequence of human PRPS1 was aligned with sequences from Opisthokonta PRPS1, Amoebozoa PrsA, Opisthokonta PRPSAP2, jawed Vertebrata PRPSAP1, Amoebozoa PrsB and eukaryotic Class II PRPS, and the corresponding allosteric site II residues were selected for generating the WebLogo similar to (B).

Supplementary Table 1. Comparison of active site residues of *B. subtilis* PRPS with corresponding amino acid residues of other Class I PRPS homologs

Bacteria		Archaea	CRuMs		Apusozoa		Amoebozoa				Opisthokonta			
B. subtilis[a]	*E. coli*	*M. jannaschii*	*D. rotans*		*T. trahens*		*R. potamoides*		*D. discoideum*		*H. sapiens*			
PRPS	PRPS	PRPS	PRPS	AP-like	PRPS	AP-like	PrsA	PrsB	PrsA	PrsB	PRPS1	PRPS2	PRPSAP1	PRPSAP2
FLAG region														
F40	F35	F32	F36	**H36**	F39	F41	F36	F40	F36	F37	F35	F35	**E42**	**E42**
D42	D37	D34	D38	D38	D41	D43	**N38**	**N42**	**N38**	**N39**	**N37**	**N37**	**N44**	**N44**
E44	E39	E36	E40	E40	E43	E45	E40	E44	E40	E41	E39	E39	E46	E46
V45	V40	I37	C41	L41	**R44**	I46	**T41**	**T45**	**T41**	**T42**	**T40**	**T40**	**T47**	**T47**
Regulatory flexible loop and ATP phosphate chain binding														
Y99	Y94	Y90	Y95	Y95	Y99	Y101	Y96	Y100	Y96	Y97	Y94	Y94	Y101	Y101
T113	T119	S104	S109	S109	S113	**A115**	T110	T114	T110	**F111**	S108	S108	**V114**	**V114**
R101	R96	R92	R97	R97	R101	R102	R98	R102	R98	R99	R96	R96	K103	K103
Q102	Q97	Q93	Q98	Q98	**A102**	Q103	Q99	Q103	Q99	Q100	Q97	Q97	Q104	Q104
D103	D98	D94	D99	D99	D103	D104	D100	**S104**	D100	**S101**	D98	D98	**S105**	**C105**
R104	**D99**	K95	R100	R100	R104	**T105**	K101	K105	K102	K102	K99	K99	K106	K106
H135	H131	H125	H131	H131	H135	H137	H132	H136	H132	H133	H130	H130	H136	H136
Pentose phosphate loop (PP loop)														
D174	D170	D163	D171	D176	D177	**S182**	D171	**G179**	D171	**G177**	D171	D171	**S177**	**S177**
G176	G172	G165	G173	G178	G179	**K184**	G173	G181	G173	G179	G173	G173	**D179**	**A179**
G177	G173	A166	G174	**S179**	G180	A185	G174	G182	G174	G180	G174	G174	A180	**S180**
Catalytic flexible loop														
K197	K194	K186	K200	K199	K205	K210	K194	R202	K196	R200	K194	K194	**G200**	**G200**
R199	R196	K188	R202	**S200**	R207	**V212**	R196	**N204**	R196	**N202**	R196	R196	**A202**	**A202**
N203	N200	**T192**	**G206**	**T205**	**G211**	**Q216**	N200	**L208**	N200	**S206**	N200	N200	**E206**	**E206**
Ribose-5-phosphate binding loop														
D223	D220	D212	D228	D306	D233	D254	D220	D258	D200	D235	D220	D220	D256	D257
D224	D221	D213	D229	D307	D234	D255	D221	D259	D221	D236	D221	D221	D257	D258
D227	D224	**S216**	D232	D310	D237	**F258**	D224	D262	D224	D239	D224	D224	D260	D261
T228	T225	T217	T233	T311	T238	S259	T225	T263	T225	T240	T225	T225	**D261**	**D262**
A229	G226	G218	A234	G312	C239	G260	C226	C264	C226	C241	C226	C226	V262	V263
T231	T228	T220	T236	T314	T241	T262	T228	T266	T228	T243	T228	T228	S264	S265
I232	L229	M221	L237	I315	L242	L263	L229	L267	L229	L244	I229	I229	F265	F266

[a]The annotated amino acid residues of the active site of *B. subtilis* PRPS enzyme are listed based on their functions. Amino acid residues at similar positions based on pairwise alignment in PRPS of *E. coli* (Accession #U00096) ; PRPS of *M. jannaschii* (Accession #L77117); PRPS (annotated from SRX3153023) and PRPSAP-like (annotated from SRX3153023) of *D. rotans*; PRPS (XP_013753676.1) and PRPSAP-like (XP_013760460.1) of *T. trahens*; PrsA (annotated from SRX8374346-9) and PrsB (annotated from SRX8374346-9) of *R. potamoides*; PrsA (XP_638466.1)

and PrsB (XP_645319.1) of *D. discoideum*; PRPS1 (NP_002755.1), PRPS2 (NP_002756.1), PRPSAP1 (AAH09012.1), and PRPSAP2 (NP_001340030.1) of human are shown. Non-conserved residues relative to *B. subtilis* PRPS enzyme are represented in bold. Active site residues appear to be poorly conserved in PRPSAP-like, PrsB, PRPSAP1 and PRPSAP2 proteins.
