# Supplementary material for: The role of gene duplication and paralog specialisation in the evolution of the mammalian PRPS complex
Source: Nat Commun. 2025 Jul 8;16:6076. doi: 10.1038/s41467-025-61216-z (PMC12238573; doi:10.1038/s41467-025-61216-z)
Supplement: Supplementary file 3 — Description of Additional Supplementary Files [file 41467_2025_61216_MOESM3_ESM.pdf]

Description of Additional Supplementary Files

File Name: Supplementary Data 1

Description: Bacterial and Eukaryotic Class I PRPS sequences

File Name: Supplementary Data 2

Description: Bacterial and Eukaryotic Class II PRPS sequences

File Name: Supplementary Data 3

Description: Extended information for splicing analysis (Fig. 1B)

File Name: Supplementary Data 4

Description: Affinity purification of PRPS-GFP in cultured cells (NIH3T3 and HEK293T)- LC-MS/MS

File Name: Supplementary Data 5

Description: Affinity purification of PRPSAP-GFP in cultured cells (NIH3T3 and HEK293T)- LC-MS/MS

File Name: Supplementary Data 6

Description: Identification of high molecular weight protein/protein complexes in human cultured cells (HEK293T) LC-MS/MS

File Name: Supplementary Data 7

Description: Untargeted NMR-metabolomic study in NIH3T3 (mouse embryonic fibroblasts)

File Name: Supplementary Data 8

Description: Oligos used in CRISPR knock-out and knock-ins
